# Hematological Indices Are Useful in Predicting Complications of Liver Cirrhosis

**DOI:** 10.3390/jcm12144820

**Published:** 2023-07-21

**Authors:** Tijana Glisic, Dusan D. Popovic, Iva Lolic, Aleksandar Toplicanin, Katarina Jankovic, Sanja Dragasevic, Marko Aleksic, Mihailo Stjepanovic, Branislav Oluic, Vera Matovic Zaric, Mirjana M. Radisavljevic, Milica Stojkovic Lalosevic

**Affiliations:** 1 Clinic of Gastroenterology and Hepatology, University Clinical Center of Serbia, 11000 Belgrade, Serbia; loliciva@gmail.com (I.L.); aleksandartoplicanin4@gmail.com (A.T.); kayajan91@gmail.com (K.J.); dragasevicsanja@gmail.com (S.D.); sloncemajmunce@gmail.com (V.M.Z.); 2 Faculty of Medicine, University of Belgrade, 11000 Belgrade, Serbia; pduschan@gmail.com (D.D.P.); marko.aleksich@gmail.com (M.A.); mihailo.stjepanovic@gmail.com (M.S.); baneoluic@gmail.com (B.O.); 3 Department of Gastroenterology, Clinical and Hospital Center “Dr Dragisa Misovic-Dedinje”, 11000 Belgrade, Serbia; 4 Clinic for Gastroenterohepatology, University Clinical Center Nis, 18000 Nis, Serbia; mirjana2409@yahoo.com

**Keywords:** liver cirrhosis, non-invasive scores, hematological indices

## Abstract

Background: Liver cirrhosis is the final stage of chronic liver disease. We aimed to evaluate non-invasive scores as predictors of complications and outcome in cirrhotic patients. Methods: A total of 150 cirrhotic patients were included. Models for end-stage liver disease (MELD), albumin-bilirubin (ALBI) score, neutrophil-lymphocyte ratio (NLR), monocyte-lymphocyte ratio (MoLR), and neutrophil-lymphocyte-albumin ratio (NLA) scores were tested in relation to the development of complications and mortality using receiver operating characteristic (ROC) curves. Results: The ROC curve analysis showed (area under the curve) AUC values of NLR, NLA, ALBI, and MELD of 0.711, 0.730, 0.627, and 0.684, respectively, for short-term mortality. MELD, ALBI, and NLA scores showed a statistically significant correlation with hepatic encephalopathy (*p* = 0.000 vs. 0.014 vs. 0.040, respectively), and the MELD cut-off value of 16 had a sensitivity of 70% and a specificity of 52% (AUC: 0.671, 95% CI (0.577–0.765)). For the assessment of the presence of ascites, the AUC values for NLA and MoLR were 0.583 and 0.658, respectively, with cut-offs of 11.38 and 0.44. Conclusions: MELD, ALBI, and NLA are reliable predictors of hepatic encephalopathy. NLA and MoLR showed a significant correlation with the presence of ascites, and MELD, ALBI, NLR, and NLA have prognostic value to predict 30-day mortality in cirrhotic patients.

## 1. Introduction

Liver cirrhosis is defined as the final stage of all chronic liver diseases. Cirrhosis currently causes 1.16 million deaths Worldwide, making it the 11th most common cause of death.

It is well known that the main characteristic of liver cirrhosis is a diffuse hepatic process characterized by fibrosis and the irreversible conversion of normal liver architecture into structurally abnormal nodules as a response to liver injury. The major causes of mortality in patients with liver cirrhosis are complications [1,2]. Cirrhosis-related portal hypertension (PH) leads to the formation of collateral channels and the expansion of plasma volume due to the activation of the renin-angiotensin-aldosterone axis, which alters systemic hemodynamics and forms the basis of complications in cirrhosis. Additionally, cirrhosis is associated with immune dysfunction and is characterized by a combination of systemic inflammation and an immune deficiency state.

A possible contributing factor in systemic inflammation is abnormal bacterial translocation, which further contributes to the development of circulatory dysfunction and failure. Systemic inflammation is related to alterations in peripheral blood leukocytes, which are reflected in the neutrophil-to-lymphocyte ratio (NLR) [3].

NLR is an inexpensive and easily available marker of systemic inflammatory response. Moreover, NRL significantly correlates with advanced inflammation in different diseases and conditions: alcoholic hepatitis, non-alcoholic fatty liver disease (NAFLD), and type 2 diabetes mellitus. Furthermore, numerous hematological indices derived from routine hemogram tests have been suggested as novel markers of inflammation. Red cell distribution width (RDW) and mean platelet volume (MPV) have been shown to increase in patients with irritable bowel syndrome (IBS) [4]. Other hemogram markers such as platelet distribution width (PDW), neutrophil to lymphocyte ratio (NLR), platelet to lymphocyte ratio (PLR), and monocyte to lymphocyte ratio (MoLR) could serve as potential predictors of inflammation in diseases such as type 2 diabetes mellitus, chronic obstructive pulmonary disease, cancer, rheumatoid arthritis, and NAFLD [5,6,7,8,9,10,11]. Previous studies showed that these previously mentioned parameters can be applied in the assessment of liver disorders [12,13,14,15]. In the majority of studies, NLR has been explored in the assessment of liver fibrosis as well as in predicting the outcomes of cirrhotic patients [16].

NLR is a great reflector of immunity. Namely, it addresses both innate and adaptive immune responses [17]. Neutrophils have an influence on immune pathways, first by inducing the production of chemokines and cytokines [18,19]. A rise in neutrophil count and further increase in NLR may be observed in various conditions [20,21,22,23,24,25,26,27]. This is explained by the severe inflammation seen in these conditions, which emphasizes the accumulation of different inflammatory cells, including neutrophils. Moreover, in previous studies, NLR has been identified as a potential predictor of mortality in the general population. Namely, in the study, XY NLR was significantly associated with higher overall mortality [17]. The Rotterdam study revealed that NLR levels are related to higher overall mortality [28].

The major histocompatibility complex (MHC) controls lymphocyte differentiation [29]. In the study of Meng et al., it has been shown that parameters of systemic inflammation reflect the severity of hepatitis virus infection [13]. Another study of chronic hepatitis B virus (HBV) infection patients indicated that NLR and PLR were significantly correlated with the levels of serum HBV-DNA and HBeAg [12].

Systemic inflammation has a significant role in the pathogenesis of various liver diseases. Several serum markers have been studied as a prognostic guide for predicting outcome and improving management of decompensated liver disease. Bearing this in mind, Zhang et al., designed a new model called neutrophil to lymphocyte to albumin ratio (NLA), which is calculated by combining NLR with albumin (ALB) level. It is calculated as 100 × NLR/ALB. They showed the indicator NLA can better predict patients’ 30-day mortality than the MELD in alcoholic cirrhosis patients [30].

Based on these considerations, the present study aimed to investigate whether NLR, PLR, PWR, MoLR, and NLA could serve as predictive markers for short-term mortality (30 days) and whether these markers could have a prognostic value in the development of liver cirrhosis complications. Moreover, it appears to be the first study on Serbian cirrhotic patients assessing the relationships between hematological markers, serological indices, and scoring systems in patients with liver cirrhosis.

## 2. Materials and Methods

This study was conducted in the emergency center of the University Clinical Center of Serbia on 150 hospital-treated patients from 2020–2022. Patients diagnosed with decompensated liver cirrhosis based on clinical data, laboratory tests, liver imaging, and/or histological reports enrolled in the study. Also, patients underwent both laboratory tests, ultrasonography, and endoscopic examinations. Subjects with a diagnosis of malignant tumor, age under 18 years, prior hepatic operation or splenectomy, prior transjugular intrahepatic portosystemic shunt, thrombosis of any part of the portal venous system, current or previous history of lymphoproliferative diseases, and patients with sepsis or any other focus of infection or inflammation were excluded from the study. The study was in accordance with the regulations of The Ethic Committee of our institution (Approval number: 602/2). The study was conducted according to the principles of the Helsinki Declaration (1989). Written informed consent was obtained from patients included in the study.

### 2.1. Data Collection

The data were collected through electronic medical records. Age, sex, etiology of liver diseases, ascites, hepatic encephalopathy (HE), presence of upper gastrointestinal bleeding (UGIB), endoscopic findings, red blood cells (RBC), hemoglobin (Hb), white blood cells (WBC), platelets (PLT), alanine aminotransferase (ALT), aspartate aminotransferase (AST), alkaline phosphatase (ALP), γ-glutamine transferase (GGT), international normalized ratio (INR), albumin (ALB), total bilirubin (TBIL), blood urea nitrogen (BUN), and creatinine (Cr) were recorded from the patients’ files and database of the institution. Additionally, we calculated the model for end-stage liver disease (MELD) [31], The Child–Turcotte–Pugh (CTP) [32], the albumin-bilirubin (ALBI) score [33], the neutrophil to lymphocyte ratio (NLR) [3], the platelet to lymphocyte ratio (PLR) [12], the monocyte to lymphocyte ratio (MoLR) [11], the gamma-glutamyl transpeptidase to platelets ratio (GPR) [34], and the neutrophil to lymphocyte to albumin ratio (NLA) [30].

Based on etiology, patients were divided into groups with alcoholic cirrhosis, chronic hepatitis B virus (HBV) and hepatitis C virus (HCV) infection, and autoimmune-related and cryptogenic liver cirrhosis. Patients with metabolic-associated fatty liver disease (MAFLD), which is one of the most common causes of liver disease worldwide, were also included in the group with cryptogenic cirrhosis. Decompensated cirrhosis was defined by the development of ascites, gastrointestinal hemorrhage, esophageal varices, hepatic encephalopathy, or jaundice. Also, patients came to the Department of Emergency Gastroenterohepatology when decompensation occurred.

Monitoring of patients in the study group was carried out in two ways. Some of them were invited to follow-up examinations within 30 days of the initial visit to the doctor, while a certain number of them were in a very difficult condition that required prolonged hospitalization. It should be emphasized that the study group consisted of patients treated in the intensive care unit.

All scores are presented in Table 1.

### 2.2. Evaluation of Esophageal Varices

Upon admission or during hospitalization, each patient underwent upper gastrointestinal endoscopy to evaluate the presence/absence of esophageal varices (EV) as well as their classification. The severity of EVs was classified as none, mild, and moderate/severe, based on the Baveno VI consensus and the American Association for the Study of Liver Disease (AASLD) practice guidelines [35]. Straight small caliber varices were considered mild; enlarged beady varices covering less than one-third of the lumen snake-like formation considered moderate; and bead-like or nodular formations were considered severe. The diagnosis of variceal hemorrhage was established when active bleeding from an esophageal or gastric varicose vein was observed or when a sign of recent bleeding, such as red cherry spots, was observed.

### 2.3. Statistical Analysis

Statistical analysis was performed with SPSS ver. 20.0 (IBM, Chicago, IL, USA) (Student’s *t* test, Mann–Whitney test, chi square test).

Receiver operating characteristic (ROC) curves were used for the determination of sensitivity and specificity for the diagnosis of EV. Correlation was examined using Pearson’s and Spearman’s correlation tests. Logistic regression was used in order to determine the variables responsible for the development of complications. A *p* value less than 0.05 was considered statistically significant.

## 3. Results

### 3.1. Demographic Characteristics

A total of 150 patients with liver cirrhosis were enrolled in the present study. Table 2 presents demographic and laboratory data for the study population. The average age of our patients was 58.83 ± 11.79 years old, and 77% of them were male. The main etiology of cirrhosis was alcohol abuse (73%), followed by chronic infection with HCV (11%), autoimmune-related (12%), cryptogenic (13%), and others. Some patients had two different etiologies of cirrhosis. The majority of the patients had oesophageal varices (83%), and the most common were moderate types of varices (47%). Regarding the complications, ascites was the most common complication with an occurrence rate of 77.9%, while 38.7% and 16.9% of patients developed hepatic encephalopathy and gastrointestinal bleeding, respectively. At 30 days after admission, 43 (29.3%) patients had died.

No statistically significant difference was found in the mortality rate between the groups of men and women. Regarding variceal bleeding, no statistically significant difference was found with regards to mortality. Among the examined scores, MELD, NLR, NLA, PLR, and ALBI levels were significantly higher among non-survivors than survivors (*p* < 0.001 vs. 0.000 vs. 0.000 vs. 0.014 vs. 0.003, respectively) (Table 3).

### 3.2. Prognostic Markers for 30-Day Mortality in Cirrhotic Patients

**Figure 1:**
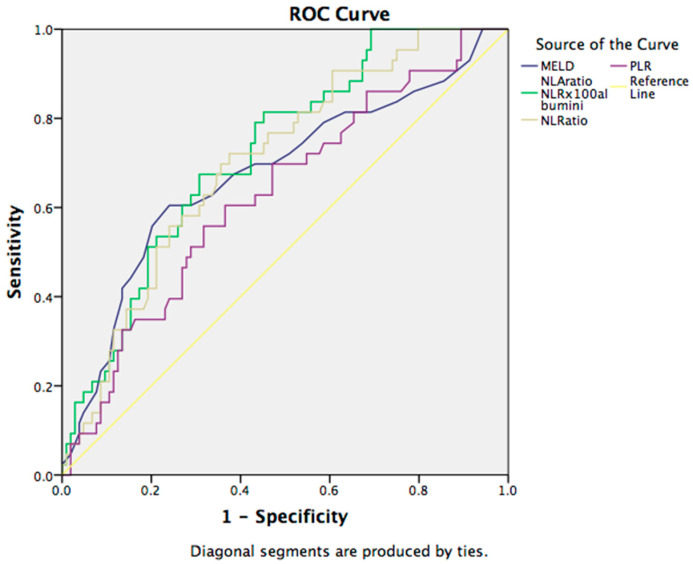
Receiver operating characteristic (ROC) curve for the discriminative ability of the prognostic scores and hematological indices to detect short-term mortality. Abbreviations: MELD—model for end-stage liver disease; NLA—neutrophil-to-lymphocyte-to-albumin ratio; NLR—neutrophil-to-lymphocyte ratio; PLR—platelets-to-lymphocyte ratio.

A ROC curve analysis was used to evaluate the sensitivity and specificity of the scores used for predicting 30-day mortality in cirrhotic patients. ROCs presenting examined parameters in patients with liver cirrhosis are presented below in Figure 1. AUC values and proposed cut-offs for MELD, NLR, NLA, and PLR in cirrhotic patients were: 0.684, 0.730, 0.711, and 0.627.

### 3.3. Prognostic Markers for the Complications in Cirrhotic Patients

Moreover, we have investigated a possible correlation between hematological biomarkers and complications of liver cirrhosis.

Our results demonstrated that on admission, 122 (81%) patients had esophageal varices, and 17% of them experienced variceal bleeding, but among the examined hematological biomarkers, none correlated statistically significantly with the presence of varices or bleeding.

**Figure 2:**
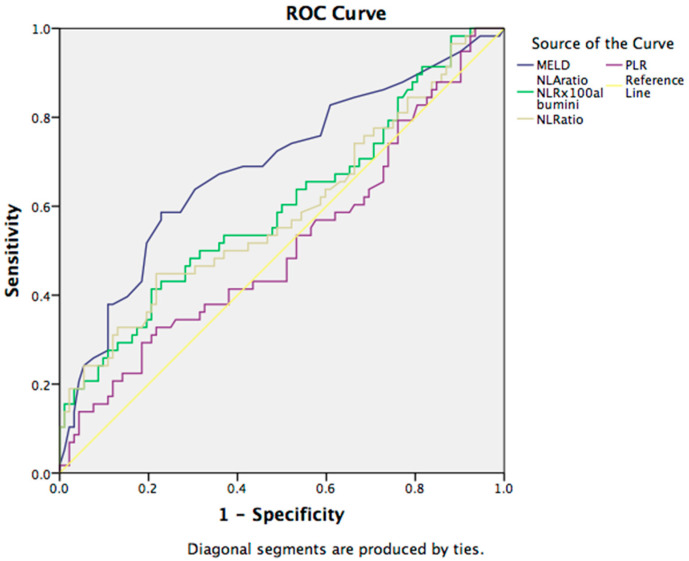
ROC curve for the discriminative ability of the prognostic scores and hematological indices to detect hepatic encephalopathy in patients with liver cirrhosis. Abbreviations: MELD—model for end-stage liver disease; NLA—neutrophil-to-lymphocyte-to-albumin ratio; NLR—neutrophil-to-lymphocyte ratio; PLR—platelets-to-lymphocyte ratio.

For the assessment of hepatic encephalopathy, ROC curve analysis showed sensitivity of 70% and specificity of 52% for the cut-off value of 16 for MELD (AUC: 0.671, 95% CI (0.577–0.765)) presented in Figure 2.

**Figure 3:**
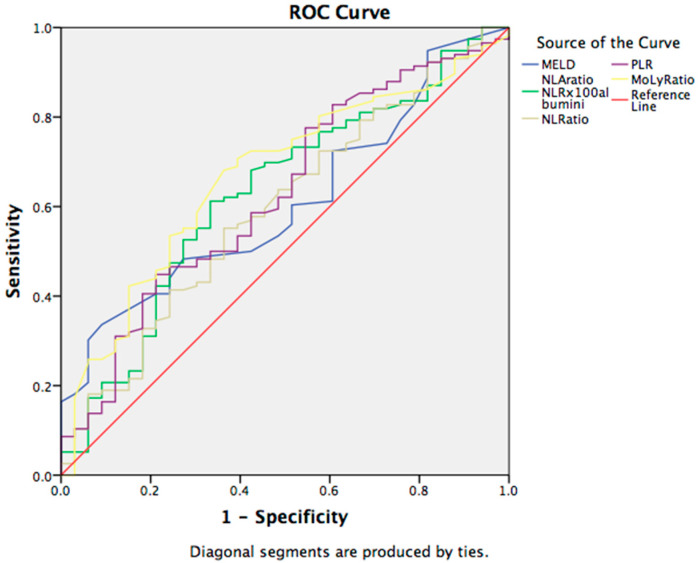
ROC curve for the discriminative ability of the prognostic scores and hematological indices to detect ascites in patients with liver cirrhosis. Abbreviations: MELD—model for end-stage liver disease; NLA—neutrophil-to-lymphocyte-to-albumin ratio; NLR—neutrophil-to-lymphocyte ratio; PLR—platelets-to-lymphocyte ratio; MoLR—monocyte-to-lymphocyte ratio; LMR—lymphocyte-to-monocyte ratio.

Additionally, we investigated the diagnostic accuracy of NLA and PLR indices for the presence of ascites in cirrhotic patients. The cut-off value of the NLA was found to be 11.38, which had a sensitivity of 70% and a specificity of 50%. A ROC curve has been provided in Figure 3.

It is interesting that MoLR showed the best predictive ability in relation to the occurrence of ascites. The AUC values and proposed cut-off for MoLR in patients with cirrhosis were for the cut-off 0.44, AUC 0.658, 95%CI 0.556–0.760, 72% sensitivity, and 58% specificity, respectively (Figure 3).

The association of the hepatic encephalopathy with survival in patients with liver cirrhosis was analyzed using logistic regression models across the groups of survivors and non-survivors. Hepatic encephalopathy was statistically significantly more prevalent in the non-survived group (*p* = 0.000).

In the current study, we found that HE associated with diabetes mellitus (DM) increases the risk of lethal outcome by more than 5.7-folds, while HE, DM, and alcohol consumption were associated with a more than 5.9-fold risk of 30-day mortality in patients with cirrhosis.

Also, hepatorenal syndrome as a complication in cirrhotic patients was statistically significantly more present in the non-survived group (*p* = 0.003), and 4-fold increases the risk for lethal outcomes.

We found a statistically significant correlation between the MELD, ALBI, and NLA scores and the presence of HE in the study group (*p* = 0.000 vs. 0.014 vs. 0.04, respectively). Additionally, the examined tests, ALBI, NLA, MoLR, LMR, and MPR, were statistically significantly correlated with the presence of ascites in the study population (*p* = 0.01 vs. 0.02 vs. 0.003 vs. 0.000 vs. 0.026, respectively), and MELD, NLA, NLR, and ALBI showed a statistically significant correlation with outcome in cirrhotic patients (*p* = 0.002 vs. 0.000 vs. 0.000 vs. 0.015, respectively) (Table 4).

## 4. Discussion

In this retrospective study, we have analyzed the association between different non-invasive scores and hematological indices, the occurrence of complications, and the outcome of patients with liver cirrhosis.

Cirrhotic patients demonstrate abnormalities in hematological indices due to the pathophysiology of the disease itself, so anemia, thrombocytopenia, and leukopenia are often seen. The pathogenesis of pancytopenia is complex and multifactorial, with portal hypertension-induced sequestration and bone marrow suppression accounting for most of the cases [36]. Previous investigations have suggested that WBC could solely serve as an independent predictor of short-term mortality in patients with acute or chronic liver failure [37].

The results of our analyses showed that the MELD, NLR, NLA, PLR, and ALBI scores in the group of non-survivors were significantly higher compared to those of the patients that survived, which is similar to the results of Maccali et al. [38] and Zhang et al. [30]. Although NLR was predominantly studied in the population of patients with liver damage, the data on the strength of the PLR score in predicting mortality in patients with cirrhosis are rather modest. PLR was mostly explored among chronic HBV/HCV patients [39,40] and patients with HIV/HCV coinfection [41]. Lower values of PLR are observed in more advanced liver fibrosis, while high levels were noted in patients with hepatocellular disease [42,43].

Although an imperative goal is to find a prognostic tool for assessing mortality in patients with liver cirrhosis, we believe that it is extremely important to find a potential marker for the development of possible complications in these patients.

When we analyzed the association of non-invasive scores in the prediction of hepatic encephalopathy, we found that there was a statistically significant association of MELD, ALBI, and NLA scores with HE. We did not find data on the predictive power of the MELD score in relation to the HE, but its impact on survival among patients with cirrhosis is well established [44,45]. Zhang et al. [32] showed that the NLR and albumin levels are significantly correlated with disease progression and survival in patients with alcohol cirrhosis. A new index—NLA, combining the NLR with albumin—can better predict patients’ 30-day mortality than the MELD, which is similar to our findings. Considering that the albumin value participates in the ALBI and NLA scores, the conclusion is drawn that the albumin value has a significant influence on the development of this complication.

The diagnostic accuracy of NLA, ALBI, and PLR indices for the presence of ascites in cirrhotic patients is very high, but it is more interesting that LMR, MoLR, and MPR showed the best predictive ability in relation to the occurrence of ascites. Michalak et al. [46] demonstrated that MPR and PLR are closely related to the markers of liver fibrosis and, moreover, seem to correlate with the clinical progression of liver cirrhosis. The study of Pomacu et al. [47] showed that NLR, MoLR, and PLR are easy-to-perform and accurate biomarkers associated with liver inflammatory status, even if they did not show a significant correlation with all oxidative stress markers assessed. MoLR is a relatively novel inflammatory index that has been studied in various conditions, like gastrointestinal stromal tumor [48] and gouty arthritis [49], but Aktas et al. [50] demonstrated that MoLR in the patients with NAFLD was significantly higher than in the healthy population. However, our results have not found an association of this index with other prognostic scores or the development of complications or mortality in patients with liver cirrhosis (LC).

The present study evaluated the performance of the non-invasive scores in predicting in-hospital mortality. MELD, NLR, NLA, and ALBI strongly correlated with short-term mortality, and NLR and NLA showed the strongest correlation among the examined scores. The results of our study are consistent with the previous findings related to the NLR and NLA and their correlation to mortality risk [30,51,52].

Additionally, we determined that HE is significantly more often present in the group of patients with poor outcomes. Furthermore, patients with HE who had DM as a comorbidity were at a 5.7-fold higher risk of death. Moreover, in the current study, we have found that HE associated with DM in patients with alcoholic LC had a 5.90-fold risk of poor outcome. A possible explanation for these findings lies in mechanisms associated with MAFLD. Namely, it has been shown that DM promotes liver fibrosis and inflammation. by increasing the mitochondrial oxidative stress caused by excess triglycerides, resulting in free radical and peroxisome release and further inflammation [52,53]. Adipokines, such as leptin and tumor necrosis factor-α (TNF-α), are produced in excess [54], and deficient adiponectin (a regulatory adipokine) enables an inflammatory adipokine environment [55]. Ultimately, hepatic stellate cells (HSCs) are activated, boosting collagen production, connective tissue growth factor, and extracellular matrix, which then promote fibrosis and cirrhosis [56,57]. In a study conducted by Kikuchi et al., examining patients with alcoholic cirrhosis, DM was clearly implicated as a contributing factor for cirrhosis development [58]. Alcoholic liver disease and NAFLD have similar pathogenic origins and histologic features, differing in phenotypes and risk factors [59]. Initially, there is simple steatosis, which progresses to steatohepatitis and then hepatic fibrosis. In addition, alcohol-induced oxidative stress may promote DNA damage and incite cirrhosis in diabetic patients. As far as we know, no research has been conducted in our country on the role of different predictive scores, including hematological indices, in the evaluation and their usefulness in predicting outcome in liver cirrhosis patients. To our knowledge, this is the first study examining multiple scores as well as parameters of systemic inflammation in a Serbian cohort of patients with LC.

### Limitation of the Study

The study participants were cirrhotic patients with different etiologies of decompensated liver cirrhosis. Secondly, we did not separate patients into groups according to the degree of NLR. Third, the data presented here were from our single center, and the representativeness of this data might be limited. Fourth, in our study, the NLR was calculated only once at admission. Serial monitoring of NLR would be necessary to diagnose complications during a hospital stay.

## 5. Conclusions

In conclusion, our results suggest that MELD, ALBI, and NLA could be useful in predicting hepatic encephalopathy in patients with liver cirrhosis. ALBI, but much more NLA, MoLR, LMR, and MPR, showed a significant correlation with the presence of ascites in patients with liver cirrhosis. Also, we have found that the MELD, ALBI, NLR, and new biomarker NLA could be useful as prognostic markers of 30-day mortality in the studied cohort. Additionally, patients with hepatic encephalopathy, alcohol use, and DM, as comorbidities, are shown as an increased risk group for lethal outcome. However, further prospective studies are required to better elucidate the relationship between the prognostic scores, the development of complications of liver cirrhosis, and patient mortality due to liver disease.

## Figures and Tables

**Table 1:** Calculation of non-invasive scores and ratios.

MELD score = 9.57 × ln(Cr) + 3.78 × ln(TBIL) + 11.2 × ln(INR) + 6.43
ALBI = ((log10 bilirubin × 0.66) + (albumin × (−0.085)))
NLR = Neu/Ly
PLR = Plt/Ly
MoLR = Mo/Ly
GPR = GGT/Plt
NLA = NLR/albumin

Abbreviations: MELD—model for end-stage liver disease; Cr—creatinine; TBIL—total serum bilirubin; INR—international normalized ratio; ALBI—albumin–bilirubin; NLR—neutrophil-to-lymphocyte ratio; Neu—neutrophils; Ly—lymphocytes; PLR—platelets-to-lymphocyte ratio; Plt—platelets; MoLR—monocyte to lymphocyte ratio; Mo—monocytes; GGT—gamma-glutamyl transpeptidase; GPR—gamma-glutamyl transpeptidase to platelets ratio; NLA—neutrophil-to-lymphocyte-to-albumin ratio.

**Table 2:** Clinical characteristics and laboratory data in patients with liver cirrhosis.

Variables	Total Patients (n = 150)
Sex (m/f)	116/34
Age (years)	58.83 ± 11.79
Etiology	
Alcohol	109
Hepatitis B	3
Hepatitis C	17
Autoimmune disease	18
Wilson disease	3
Toxic	0
Cryptogenic DM	20 39
Laboratory test	
Hg (g/L) ^a^	96.17 ± 27.24
WBC (10^9^/L) ^a^ Lymphocytes Neutrophiles Eosinophiles Monocytes	8.98 ± 5.28 1.24 6.81 0.12 0.7
PLT (10^9^/L) ^a^	115.58 ± 63.83
TBIL (mmol/L) ^b^	103.25
Alb (g/L) ^b^	29.81
AST (U/L) ^b^	104.08
ALT (U/L) ^b^	44.29
ALP (U/L)^b^	134.27
GGT (U/L) ^b^	171.42
BUN (mmol/L) ^b^	15.39
Cr (µmol/L) ^b^	141.90
INR^b^	1.65
NH4 (µmol/L) ^b^	72.34
CRP (mg/L) ^b^	32.56
Pct (ng/L) ^b^	1.76
Na (mmol/L) ^b^	135.70
LDH (U/L) ^b^	554.04
Cholesterol (mmol/L)	2.97
Triglycerides (mmol/L) Esophageal varices Mild Moderate Large	1.13 117 (78.5%) 43 (28.7%) 43 (28.7%) 31 (20.7%)
MELD score ^b^	19.83
ALBI score ^b^	−1.40
NLR ^b^	7.08
NLA ^b^	25.71
PLR ^b^	111.75
MoLR ^b^	0.67
LMR ^b^	2.13
MPR ^b^	0.13
GPR ^b^	2.16

Abbreviations: ^a^ mean ± SD; ^b^ median (IQR); pts—patients; n—number of patients; DM—diabetes mellitus; Hb—hemoglobin; WBC—white blood cell; PLT—platelet; TBil—total bilirubin; Alb—albumin; AST—aspartate aminotransferase; ALT—alanine aminotransferase; ALP—alkaline phosphatase; GGT—gamma glutamine transferase; BUN—blood urea nitrogen; Cr—creatinine; INR—international normalized ratio; CRP—C-reactive protein; Pct—procalcitonin; Na—sodium; LDH—lactic acid dehydrogenase; NH4—ammonium ion; MELD—model for end stage liver disease; CTP—Child–Turcotte–Pugh; ALBI—albumin-bilirubin; NLR—neutrophil-to-lymphocyte ratio; NLA—neutrophil-to-lymphocyte-to-albumin ratio; PLR—platelets to lymphocyte ratio; MoLR—monocyte-to-lymphocyte ratio; LMR—lymphocyte-to-monocyte ratio; MPR—mean platelet volume-to-platelets ratio; GPR—gamma-glutamine transferase-to-platelets ratio.

**Table 3:** Clinical characteristics, complications, and score values between the survived and non-survived groups of patients with cirrhosis.

Variables	Survived pts	Non-Survived pts	*p* Value
Sex (m/f)	75/25	36/7	0.924
Variceal bleeding	103	42	0.333
MELD	17.99	23.79	0.001
NLA	19.98	37.75	0.000
NLR	5.95	9.52	0.000
PLR	103.84	127.91	0.014
MoLR	0.642	0.758	0.192
LMR	2.21	1.97	0.171
MPR	0.130	0.121	0.213
GPR	2.08	2.46	0.984
ALBI	−1.51	−1.15	0.003

Abbreviations: MELD—model for end stage liver disease; NLA—neutrophil-to-lymphocyte-to-albumin ratio; NLR—neutrophil-to-lymphocyte ratio; PLR—platelets-to-lymphocyte ratio; MoLR—monocyte-to-lymphocyte ratio; LMR—lymphocyte-to-monocyte ratio; MPR—mean platelet volume-to-platelets ratio; GPR—gamma-glutamine transferase-to-platelets ratio; ALBI—albumin-bilirubin ratio.

**Table 4:** Values of non-invasive scores and hematological indices in relation to the presence of hepatic encephalopathy, ascites, and outcome in patients with liver cirrhosis.

Variables	Hepatic Encephalopathy			Ascites			Outcome		
	Yes	No	*p* Value	Yes	No	*p* Value	Live	Dead	*p* Value
MELD	23.55	17.48	0.000	20.72	17.03	0.056	17.99	23.79	0.001
NLA	35.08	19.79	0.014	28.02	18.09	0.011	19.98	37.75	0.000
NLR	9.24	5.72	0.040	7.52	5.67	0.022	5.95	9.52	0.000
PLR	121.67	105.49	0.065	118.32	89.55	0.092	103.84	127.91	0.014
MoLR	0.79	0.60	0.760	0.73	0.52	0.025	0.642	0.758	0.192
LMR	2.02	2.20	0.173	1.94	2.71	0.003	2.21	1.97	0.171
MPR	0.13	0.13	0.175	0.12	0.14	0.000	0.130	0.121	0.213
GPR	2.56	1.90	0.931	2.08	2.46	0.026	2.08	2.46	0.984
ALBI	−1.25	−1.50	0.063	−1.32	−1.68	0.909	−1.51	−1.15	0.003

Abbreviations: MELD—model for end stage liver disease; ALBI—albumin–bilirubin; NLA—neutrophil-to-lymphocyte-to-albumin ratio; NLR—neutrophil-to-lymphocyte ratio; PLR—platelets-to-lymphocyte ratio; MoLR—monocyte-to-lymphocyte ratio; LMR—lymphocyte-to-monocyte ratio; MPR—mean platelet volume-to-platelets ratio; GPR—gamma-glutamine transferase-to-platelets ratio.

## Data Availability

All data are available upon request from corresponding authors.
